# Metabarcoding data of mitochondrial cytochrome *c* oxidase subunit 1 gene from bulk community of aquatic organisms collected from Nara Prefecture, Japan

**DOI:** 10.1016/j.dib.2022.108599

**Published:** 2022-09-15

**Authors:** Kei Wakimura, Koji Inai, Kazumi Tanida, Kozo Watanabe, Mikio Kato

**Affiliations:** a Faculty of Liberal Arts and Sciences, Osaka Metropolitan University - Nakamozu Campus, 1-1 Gakuencho, Sakai 599-8531, Japan; b Department of Biology, Graduate School of Science, Osaka Metropolitan University - Nakamozu Campus, 1-1 Gakuencho, Sakai 599-8531, Japan; c Osaka Museum of Natural History, Nagai Park, Higashi-sumiyoshi, Osaka, 546-0034, Japan; d Center for Marine Environment Studies, Ehime University, Bunkyo-cho 3, Matsuyama 790-8577, Japan

**Keywords:** Benthic fauna, Cytochrome *c* oxidase subunit 1 gene (*cox1*), Metabarcoding, Riverine metagenome, Species identification

## Abstract

Riverine metabarcoding data were obtained from the Takamigawa River, a tributary of the Kinokawa River, in Nara Prefecture (Central Honshu, Japan). We extracted DNA from bulk community samples of aquatic organisms, most of which could not be morphologically identified at species level due to their small body size (0.12 - 2 mm length). A partial coding region of the mitochondrial cytochrome *c* oxidase subunit 1 gene (*cox1*) was amplified using PCR, and the amplicon was subjected to high-throughput parallel sequencing (Illumina MiSeq). The 313 bp paired-end sequence reads were classified into operational taxonomic units (OTUs), their species boundaries were delineated using the Generalised Mixed Yule Coalescent (GMYC) method, and taxonomic names of the GMYC species were assigned using basic local alignment search tool (BLAST) against International DNA Databases (INSD: GenBank, ENA, and DDBJ).

**Specifications Table**

**Table:** 

Subject:	Biodiversity
Specific subject area:	DNA taxonomy and species identification of stream insects
Type of data:	Figure, Table, and amplicon sequence data
Data acquisition:	Illumina MiSeq platform, MrBayes 3.2.7a, SPLITS package in R
Data format:	Raw sequence reads Analysed metabarcoding data
Description of data collection:	DNA extraction of three bulk community samples of aquatic organisms, amplicon sequencing of *cox1*, quality-filtering of sequence reads, operational taxonomic units (OTUs) clustering of sequence reads using *ad hoc* R script, building a phylogenetic tree of OTUs using Markov chain Monte Carlo (MCMC) by MrBayes 3.2.7a with the GTR+I+G molecular clock model, estimation of taxonomic boundaries among OTUs based on GMYC approach using SPLITS package in R, and BLAST search against INSD.
Data source location:	Bulk community samples of aquatic organisms collected from Takamigawa River, Nara Prefecture, Central Honshu, Japan. Sampling site: Aridoshi (ARD), 34°23′23″N 135°59′17″E Sampling site: Shisetsu at Kozugawa (SST), 34°23′53″N 135°59′34″E
Data accessibility:	With the article: Phylogenetic tree, taxonomy, abundance of the OTUs. In a public repository: DNA Databank of Japan (DDBJ) and Mendeley Data. The sequencing data are available in DDBJ under the SRA ID: DRA012522; BioProject No. PRJDB11912; BioSample: SAMD00388525, SAMD00388526, SAMD00388527, SAMD00388528, SAMD00388529, SAMD00388530, SAMD00388531, SAMD00388532; Run: DRR311861, DRR311862, DRR311863, DRR311864, DRR311865, DRR311866, DRR311867, DRR311868. OTU sequences in FASTA format and an R script for clustering OTUs are deposited in Mendeley Data with DOI:10.17632/pfhf4kt37s.1

## Value of the Data

• The metabarcoding result enables us to evaluate the efficacy of DNA-based biodiversity assessment in comparison with conventional morphology-based species identification data collected from the same localities.
• The new OTU clustering algorism that compare the read abundances between neighbouring OTUs is available as an R script, which could be used widely to process amplicon sequence data.
• The results are beneficial for researchers working on both DNA metabarcoding and conventional morphology-based faunal surveys.
• The data will serve as a reference barcode sequence data of benthic fauna in mountain streams in the Central Honshu, Japan.

## 1 Data Description

Raw sequence reads of partial coding regions of *cox1* in riverine metagenomes are deposited in DNA Data Bank of Japan (DDBJ). DDBJ accession numbers DRR311861 and DRR311862 were obtained from the run environment (slow riffle) of Aridoshi (ARD), DRR311863-DRR311865 from the rapid environment of Shisetsu (SST), and DDR311866-DRR311868 from the run environment of Shisetsu (SST). There were 472,697 reads in total of DRR311861- DRR311868, and they were classified into 888 OTUs.

Phylogenetic tree of the 888 OTUs was reconstructed by MrBayes 3.2.7a based on the GTR+I+G molecular clock model, and the species boundaries were delineated using the Generalised Mixed Yule Coalescent (GMYC) method (Fig. 1). A total of 369 GMYC species were presumed from 888 OTUs.

Representative sequences of the GMYC species (the most abundant OTUs in the species clusters; marked with GMYC IDs in Fig. 1) were queried on INSD, and the sequence data showing the highest similarity were retrieved (Table 1). According to the critical similarity scores for species identification [1], OTUs showing similarity scores (percent identity) greater than 95% are considered to be properly deduced. In the present results, 137 out of 369 GMYC species were assigned to INSD with >95% similarity. The DNA barcode references of the remaining unassigned GMYC species may not have been registered in INSD yet. These unassigned GMYC species can be extrapolated to coarser level taxonomic names (i.e., genus, family, or order) according to the critical similarity scores proposed by Inai et al. [1].

**Table 1:** List of INSD data showing highest similarity with representative OTUs assigned as species by GMYC.

GMYC ID	(Number of identical sites/ query size)	Similarity scores (% identity)	INSD accession number Organism name/ Family/ Order	Remark on taxon
10	(313/313)	100.0	MK774296 Paracinygmula zhiltzovae / Heptageniidae / Ephemeroptera	
11	(313/313)	100.0	KP970686 Epeorus aesculus / Heptageniidae / Ephemeroptera	
12	(313/313)	100.0	MK774388 Epeorus curvatulus / Heptageniidae / Ephemeroptera	
15	(313/313)	100.0	JQ655115 Epeorus latifolium / Heptageniidae / Ephemeroptera	
17	(313/313)	100.0	MK774354 Epeorus latifolium / Heptageniidae / Ephemeroptera	
18	(313/313)	100.0	LC513137 Afronurus yoshidae / Heptageniidae / Ephemeroptera	Ecdyonurus yoshidae Takahashi, 1924
20	(313/313)	100.0	LC106815 Isonychia japonica / Isonychiidae / Ephemeroptera	
22	(313/313)	100.0	GU354162 Cinygmula sp. MK-2010 / Heptageniidae / Ephemeroptera	
25	(313/313)	100.0	MK774360 Drunella trispina / Ephemerellidae / Ephemeroptera	
27	(313/313)	100.0	MK774334 Drunella ishiyamana / Ephemerellidae / Ephemeroptera	
34	(313/313)	100.0	LC489696 Dipteromimus tipuliformis / Dipteromimidae / Ephemeroptera	
38	(313/313)	100.0	GU354161 Paraleptophlebia chocolata / Leptophlebiidae / Ephemeroptera	
39	(313/313)	100.0	MN961294 Ephemera strigata / Ephemeridae / Ephemeroptera	
41	(313/313)	100.0	KP970715 Ephemerellidae sp. OPU_BS_E2014-4 / Ephemerellidae / Ephemeroptera	
66	(313/313)	100.0	MN344567 Kamimuria tibialis / Perlidae / Plecoptera	
70	(313/313)	100.0	MN961334 Neoperla sp. OPU BS 2019-094-DB-PL / Perlidae / Plecoptera	
74	(313/313)	100.0	MK774376 Paragnetina sp. OPU_BS_2017-320-YS-PL / Perlidae / Plecoptera	
76	(313/313)	100.0	AB770094 Togoperla limbata / Perlidae / Plecoptera	
77	(313/313)	100.0	MK774375 Paragnetina sp. OPU_BS_2017-319-YS-PL / Perlidae / Plecoptera	
81	(313/313)	100.0	MK492252 Perlomyia isobeae / Leuctridae / Plecoptera	
91	(313/313)	100.0	LC462303 Corynoneura celtica / Chironomidae / Diptera	
96	(313/313)	100.0	LC462341 Cricotopus metatibialis / Chironomidae / Diptera	
97	(313/313)	100.0	LC462339 Cricotopus metatibialis / Chironomidae / Diptera	
119	(313/313)	100.0	AB838600 Cricotopus bimaculatus / Chironomidae / Diptera	
126	(313/313)	100.0	LC462328 Synorthocladius tamaparvulus / Chironomidae / Diptera	
137	(313/313)	100.0	LC329260 Rheopelopia joganflava / Chironomidae / Diptera	
161	(313/313)	100.0	LC329063 Cladotanytarsus vanderwulpi / Chironomidae / Diptera	
171	(313/313)	100.0	LC342229 Tanytarsus tamaundecimus / Chironomidae / Diptera	
177	(313/313)	100.0	LC329303 Tanytarsus arduennensis / Chironomidae / Diptera	
183	(313/313)	100.0	LC462285 Microtendipes famiefeus / Chironomidae / Diptera	
185	(313/313)	100.0	LC329175 Polypedilum asakawaense / Chironomidae / Diptera	
199	(313/313)	100.0	JQ655116 Baetis sp. OPU_BS_B2011-494 / Baetidae / Ephemeroptera	
212	(313/313)	100.0	KP970716 Baetidae sp. OPU_BS_B2014-5 / Baetidae / Ephemeroptera	
213	(313/313)	100.0	KF563015 Baetiella japonica / Baetidae / Ephemeroptera	
215	(313/313)	100.0	MH260764 Stenopsyche sauteri / Stenopsychidae / Trichoptera	
216	(313/313)	100.0	KX291699 Stenopsyche marmorata / Stenopsychidae / Trichoptera	
225	(313/313)	100.0	JX448423 Parascythopus exsulans / Curculionidae / Coleoptera	Parascythopus intrusus (Kôno, 1948)
230	(313/313)	100.0	KX106254 Rhyacophila kawamurae / Rhyacophilidae / Trichoptera	
231	(313/313)	100.0	MK774340 Rhyacophila kisoensis / Rhyacophilidae / Trichoptera	
237	(313/313)	100.0	KX104086 Goera japonica / Goeridae / Trichoptera	
238	(313/313)	100.0	KX104891 Goera squamifera / Goeridae / Trichoptera #	species not recorded from Japan
240	(313/313)	100.0	HQ967404 Trichoptera sp. BOLD:AAN9649 / / Trichoptera	
246	(313/313)	100.0	LT903836 Nais communis / Naididae / Haplotaxida	Oligochaeta
255	(313/313)	100.0	KY284174 Eiseniella tetraedra / Lumbricidae / Crassiclitellata	Oligochaeta
295	(313/313)	100.0	MH260783 Cheumatopsyche infascia / Hydropsychidae / Trichoptera	
296	(313/313)	100.0	MH260789 Cheumatopsyche galloisi / Hydropsychidae / Trichoptera	
297	(313/313)	100.0	KX104427 Ceratopsyche orientalis / Hydropsychidae / Trichoptera	Hydropsyche orientalis Martynov, 1934
298	(313/313)	100.0	MK774339 Ceratopsyche setensis / Hydropsychidae / Trichoptera	Hydropsyche setensis Iwata, 1927
302	(312/312)	100.0	HQ967450 Trichoptera sp. BOLD:AAN9228 / / Trichoptera	
305	(313/313)	100.0	AP010696 Homo sapiens / Hominidae / Primates	human (contaminant?)
306	(295/295)	100.0	AB988797 Rhinogobius flumineus / Gobiidae / Gobiiformes	fish (lizard goby)
307	(301/301)	100.0	AB708313 Lestes temporalis / Lestidae / Odonata	
308	(301/301)	100.0	AB708713 Onychogomphus viridicostus / Gomphidae / Odonata	
322	(313/313)	100.0	GU722900 Hydra vulgaris / Hydridae / Anthoathecata	
337	(313/313)	100.0	MF000493 Craspedacusta sowerbii / Olindiidae / Limnomedusae	peach blossom jellyfish
351	(313/313)	100.0	JN574872 Calonectria colhounii / Nectriaceae / Hypocreales	fungus
354	(313/313)	100.0	MT010913 Fusarium globosum / Nectriaceae / Hypocreales	fungus
3	(312/313)	99.7	MK774315 Rhithrogena japonica / Heptageniidae / Ephemeroptera	
56	(312/313)	99.7	MK774371 Oyamia sp. OPU_BS_2017-315-YS-PL / Perlidae / Plecoptera	
79	(312/313)	99.7	MK132349 Amphinemura megaloba / Nemouridae / Plecoptera	
98	(312/313)	99.7	LC050960 Cricotopus metatibialis / Chironomidae / Diptera	
135	(312/313)	99.7	LC329252 Rheopelopia joganflava / Chironomidae / Diptera	
136	(312/313)	99.7	LC329258 Rheopelopia joganflava / Chironomidae / Diptera	
166	(312/313)	99.7	EF585416 Stempellinella coronata / Chironomidae / Diptera	
182	(312/313)	99.7	LC329124 Microtendipes britteni / Chironomidae / Diptera	
188	(312/313)	99.7	LC329243 Polypedilum unifascium / Chironomidae / Diptera	
223	(312/313)	99.7	LT991411 Ochthebius japonicus / Hydraenidae / Coleoptera	
235	(312/313)	99.7	MN961310 Glossosoma ussuricum / Glossosomatidae / Trichoptera	
336	(312/313)	99.7	MF177101 Craspedacusta sowerbii / Olindiidae / Limnomedusae	peach blossom jellyfish
1	(311/313)	99.4	MK774405 Rhithrogena japonica / Heptageniidae / Ephemeroptera	
8	(311/313)	99.4	MK774358 Rhithrogena tetrapunctigera / Heptageniidae / Ephemeroptera	
9	(311/313)	99.4	MN961320 Heptageniidae sp. OPU BS 2019-040-DB-EP / Heptageniidae / Ephemeroptera	
26	(311/313)	99.4	MK774398 Drunella kohnoi / Ephemerellidae / Ephemeroptera	
107	(311/313)	99.4	LC329151 Orthocladius kanii / Chironomidae / Diptera	
127	(311/313)	99.4	LC329155 Parakiefferiella tamatriangulata / Chironomidae / Diptera	
129	(311/313)	99.4	LC329157 Parametriocnemus stylatus / Chironomidae / Diptera	
179	(311/313)	99.4	LC329188 Polypedilum hiroshimaense / Chironomidae / Diptera	
194	(311/313)	99.4	MN961306 Apsilochorema sutshanum / Hydrobiosidae / Trichoptera	
196	(311/313)	99.4	MK144522 Nymphomyia alba / Nymphomyiidae / Diptera	
197	(311/313)	99.4	KP970694 Baetis sp. MK-2015d / Baetidae / Ephemeroptera	
234	(311/313)	99.4	MN961311 Glossosoma altaicum / Glossosomatidae / Trichoptera	
301	(311/313)	99.4	HQ967424 Trichoptera sp. BOLD:AAN3967 / / Trichoptera	
21	(310/313)	99.0	MK774393 Epeorus ikanonis / Heptageniidae / Ephemeroptera	
55	(310/313)	99.0	MK774343 Baetidae sp. OPU_BS_2017-141-MA-EP / Baetidae / Ephemeroptera	
123	(310/313)	99.0	LC462337 Orthocladius tamarutilus / Chironomidae / Diptera	
164	(310/313)	99.0	LC329266 Rheotanytarsus tamasecundus / Chironomidae / Diptera	
168	(310/313)	99.0	LC329147 Neozavrelia tamanona / Chironomidae / Diptera	
187	(310/313)	99.0	LC329176 Polypedilum asoprimum / Chironomidae / Diptera	
294	(310/313)	99.0	KX103761 Psychomyia nipponica / Psychomyiidae / Trichoptera	
86	(309/313)	98.7	LC462305 Corynoneura kibunelata / Chironomidae / Diptera	
95	(309/313)	98.7	LC462311 Thienemanniella majuscula / Chironomidae / Diptera	
159	(309/313)	98.7	LC329058 Cladotanytarsus vanderwulpi / Chironomidae / Diptera	
210	(309/313)	98.7	KP970709 Acentrella gnom / Baetidae / Ephemeroptera	
242	(309/313)	98.7	GQ355370 Nais variabilis / Naididae / Haplotaxida	Oligochaeta
250	(309/313)	98.7	GQ355366 Chaetogaster diaphanus / Naididae / Haplotaxida	Oligochaeta
45	(308/313)	98.4	JQ655113 Serratella setigera / Ephemerellidae / Ephemeroptera	
46	(308/313)	98.4	JQ655113 Serratella setigera / Ephemerellidae / Ephemeroptera	
85	(308/313)	98.4	LC462302 Corynoneura tenuistyla / Chironomidae / Diptera	
90	(308/313)	98.4	LC462308 Corynoneura lobata / Chironomidae / Diptera	
113	(308/313)	98.4	LC329246 C / Chironomidae / Diptera	
236	(308/313)	98.4	KX106735 Rhyacophila angulata / Rhyacophilidae / Trichoptera #	species not recorded from Japan
16	(307/313)	98.1	JQ655115 Epeorus latifolium / Heptageniidae / Ephemeroptera	
31	(307/313)	98.1	LC481971 Cincticostella elongatula / Ephemerellidae / Ephemeroptera	
92	(307/313)	98.1	LC329294 Thienemanniella flaviscutella / Chironomidae / Diptera	
103	(307/313)	98.1	LC329301 Tvetenia tamaflava / Chironomidae / Diptera	
133	(307/313)	98.1	KY497570 Simulium quinquestriatum / Simuliidae / Diptera	
53	(306/313)	97.8	LC462319 Nilotanypus dubius / Chironomidae / Diptera	
209	(306/313)	97.8	KF563060 Nigrobaetis chocoratus / Baetidae / Ephemeroptera	
224	(306/313)	97.8	KM376576 Hydrocyphon satoi / Scirtidae / Coleoptera	
29	(305/312)	97.8	LC461422 Drunella ishiyamana / Ephemerellidae / Ephemeroptera	
19	(305/313)	97.4	MK774389 Ecdyonurus sp. OPU_BS_2018-038-IN-EP / Heptageniidae / Ephemeroptera	
180	(305/313)	97.4	AB731460 Polypedilum takaoense / Chironomidae / Diptera	
244	(305/313)	97.4	LN810267 Nais bretscheri / Naididae / Haplotaxida	Oligochaeta
299	(305/313)	97.4	KX291233 Oecetis raghava / Leptoceridae / Trichoptera #	species not recorded from Japan
362	(304/312)	97.4	MK468491 Ascochyta pisi / Didymellaceae / Pleosporales	fungus
30	(304/313)	97.1	LC481981 Cincticostella orientalis / Ephemerellidae / Ephemeroptera	
118	(304/313)	97.1	KX037940 Tiphobiosis hinewai / Hydrobiosidae / Trichoptera #	species not recorded from Japan
131	(304/313)	97.1	MG551479 Simulium bidentatum / Simuliidae / Diptera	
160	(304/313)	97.1	LC329058 Cladotanytarsus vanderwulpi / Chironomidae / Diptera	
181	(304/313)	97.1	LC329088 Demicryptochironomus vulneratus / Chironomidae / Diptera	
353	(304/313)	97.1	MT123351 Calonectria ilicicola / Nectriaceae / Hypocreales	fungus
28	(303/313)	96.8	MK774334 Drunella ishiyamana / Ephemerellidae / Ephemeroptera	
200	(303/313)	96.8	JQ655116 Baetis sp. OPU_BS_B2011-494 / Baetidae / Ephemeroptera	
117	(302/313)	96.5	LC329102 Eukiefferiella chuzeoctava / Chironomidae / Diptera	
186	(302/313)	96.5	LC329204 Polypedilum parviacumen / Chironomidae / Diptera	
54	(302/314)	96.2	LC462319 Nilotanypus dubius / Chironomidae / Diptera	
33	(301/313)	96.2	MH260779 Drunella sp. OPU_BS_D2016-90 / Ephemerellidae / Ephemeroptera	
47	(301/313)	96.2	MK774379 Serratella setigera / Ephemerellidae / Ephemeroptera	
104	(301/313)	96.2	LC329301 Tvetenia tamaflava / Chironomidae / Diptera	
167	(301/313)	96.2	AM398700 Micropsectra kurobemaculata / Chironomidae / Diptera	
248	(301/313)	96.2	KY633405 Slavina appendiculata / Naididae / Haplotaxida	Oligochaeta
128	(300/312)	96.2	LC329156 Parametriocnemus stylatus / Chironomidae / Diptera	
252	(299/313)	95.5	AF534855 Pristina osborni / Naididae / Haplotaxida	Oligochaeta
71	(298/313)	95.2	MN961334 Neoperla sp. OPU BS 2019-094-DB-PL / Perlidae / Plecoptera	
87	(298/313)	95.2	LC462305 Corynoneura kibunelata / Chironomidae / Diptera	
214	(298/313)	95.2	KF563015 Baetiella japonica / Baetidae / Ephemeroptera	
233	(296/311)	95.2	KX107043 Rhyacophila brevicephala / Rhyacophilidae / Trichoptera	similarity score >95%
57	(297/313)	94.9	MK774371 Oyamia sp. OPU_BS_2017-315-YS-PL / Perlidae / Plecoptera	similarity score <95%
184	(297/313)	94.9	LC329139 Microtendipes tamaogouti / Chironomidae / Diptera	
48	(296/313)	94.6	MK774379 Serratella setigera / Ephemerellidae / Ephemeroptera	
211	(296/313)	94.6	KP970709 Acentrella gnom / Baetidae / Ephemeroptera	
122	(294/311)	94.5	LC462337 Orthocladius tamarutilus / Chironomidae / Diptera	
142	(294/312)	94.2	KX946556 Tabanus thoracinus / Tabanidae / Diptera	
58	(294/313)	93.9	MK774371 Oyamia sp. OPU_BS_2017-315-YS-PL / Perlidae / Plecoptera	
205	(277/295)	93.9	MH823348 Acentrella sibirica / Baetidae / Ephemeroptera	
339	(293/313)	93.6	KR055655 Pseudogymnoascus pannorum / Pseudeurotiaceae /	fungus
361	(293/313)	93.6	MK468491 Ascochyta pisi / Didymellaceae / Pleosporales	fungus
245	(290/310)	93.5	LN810254 Piguetiella blanci / Naididae / Haplotaxida	Oligochaeta
341	(293/314)	93.3	KR055655 Pseudogymnoascus pannorum / Pseudeurotiaceae /	fungus
61	(292/313)	93.3	AB770139 Oyamia lugubris / Perlidae / Plecoptera	
72	(292/313)	93.3	MN961334 Neoperla sp. OPU BS 2019-094-DB-PL / Perlidae / Plecoptera	
146	(292/313)	93.3	MG089252 Hexatoma sp. I13-26-02 / Limoniidae / Diptera	
218	(291/312)	93.3	HQ938480 Zaitzevia sp. BOLD:AAN5764 / Elmidae / Coleoptera	
88	(288/309)	93.2	KM928316 Chironomidae sp. BOLD:AAN5033 / Chironomidae / Diptera	
344	(267/287)	93.0	KX450332 Glarea lozoyensis / Helotiaceae / Helotiales	fungus
143	(291/313)	93.0	MK396368 Tabanus thoracinus / Tabanidae / Diptera	
145	(290/312)	92.9	MG089252 Hexatoma sp. I13-26-02 / Limoniidae / Diptera	
132	(289/311)	92.9	AY251508 Simulium grossifilum / Simuliidae / Diptera	
106	(288/310)	92.9	MF458772 Orthocladiinae sp. BAP34 / Chironomidae / Diptera	
59	(290/313)	92.7	MK774371 Oyamia sp. OPU_BS_2017-315-YS-PL / Perlidae / Plecoptera	
141	(290/313)	92.7	DQ983533 Tabanus aegrotus / Tabanidae / Diptera	
124	(286/309)	92.6	MN680434 Pseudokiefferiella sp. BOLD:AAL9436 / Chironomidae / Diptera	
40	(289/313)	92.3	LC461324 Teloganopsis punctisetae / Ephemerellidae / Ephemeroptera	
114	(289/313)	92.3	JF287767 Potthastia gaedii / Chironomidae / Diptera	
116	(289/313)	92.3	HQ941600 Orthocladius rivulorum / Chironomidae / Diptera	
144	(288/313)	92.0	KC592633 Scioniini gen. NZ sp. lerda / Tabanidae / Diptera	
102	(287/312)	92.0	MT047747 Diamesa hyperborea / Chironomidae / Diptera	
360	(287/312)	92.0	KM382246 Shiraia bambusicola / Shiraiaceae / Pleosporales	fungus
227	(285/310)	91.9	MT230864 Parachauliodes continentalis / Corydalidae / Megaloptera	
134	(283/308)	91.9	KP252587 Simulium gonzalezi / Simuliidae / Diptera	
7	(287/313)	91.7	MK774358 Rhithrogena tetrapunctigera / Heptageniidae / Ephemeroptera	
60	(287/313)	91.7	AB770139 Oyamia lugubris / Perlidae / Plecoptera	
100	(287/313)	91.7	KJ082995 Apsiphortica lini / Drosophilidae / Diptera	
125	(286/312)	91.7	MG301788 Orthocladiinae sp. BIOUG23941-E02 / Chironomidae / Diptera	
109	(284/310)	91.6	KX779818 Culex gnomatos / Culicidae / Diptera	
170	(286/313)	91.4	KT613675 Tanytarsus sp. 7XL / Chironomidae / Diptera	
99	(283/310)	91.3	LC329164C / Chironomidae / Diptera	
355	(271/297)	91.2	MK674497 Podosphaera xanthii / Erysiphaceae / Erysiphales	fungus
271	(279/306)	91.2	MN673973 Sperchon glandulosus / Sperchontidae / Trombidiformes	Acariformes
2	(285/313)	91.1	MK774405 Rhithrogena japonica / Heptageniidae / Ephemeroptera	
64	(285/313)	91.1	AB770139 Oyamia lugubris / Perlidae / Plecoptera	
65	(285/313)	91.1	AB770139 Oyamia lugubris / Perlidae / Plecoptera	
340	(284/312)	91.0	EU883400 Tetracladium apiense / / Helotiales	fungus
115	(281/309)	90.9	KC750387 Cricotopus sp. 1 MEC-2013 / Chironomidae / Diptera	
342	(279/307)	90.9	KY318514 Pseudogymnoascus destructans / Pseudeurotiaceae /	fungus
93	(286/315)	90.8	KR659696 Corynoneura sp. BOLD-2016 / Chironomidae / Diptera	
121	(285/314)	90.8	JX887678 Leucophenga saigusai / Drosophilidae / Diptera	
334	(284/313)	90.7	GU070900 invertebrate environmental sample / /	unspecified
173	(283/312)	90.7	LC329269 Stempellinella tamaseptima / Chironomidae / Diptera	
228	(283/312)	90.7	KX294695 Rhyacophila lata / Rhyacophilidae / Trichoptera	
148	(282/311)	90.7	KX771129 Drosophila nikananu / Drosophilidae / Diptera	
243	(282/311)	90.7	GQ355369 Nais elinguis / Naididae / Haplotaxida	Oligochaeta
154	(272/300)	90.7	KT116489 Gymnometriocnemus brumalis / Chironomidae / Diptera	
110	(281/310)	90.6	KF839967 Simulium callidum / Simuliidae / Diptera	
130	(280/309)	90.6	KP697234 Leucophenga sp. 7 HWC-2015 / Drosophilidae / Diptera	
139	(267/295)	90.5	JF871911 Docosia sp. BOLD:AAP4732 / Mycetophilidae / Diptera	
120	(283/313)	90.4	LC582930 Chironominae sp. 11 NK-2020 / Chironomidae / Diptera	
352	(283/313)	90.4	JN574872 Calonectria colhounii / Nectriaceae / Hypocreales	fungus
101	(282/312)	90.4	MG308407 Gymnometriocnemus sp. BIOUG01460-H10 / Chironomidae / Diptera	
105	(282/312)	90.4	KY841662 Chironomidae sp. BIOUG02204-H08 / Chironomidae / Diptera	
94	(281/311)	90.4	MF825573 Chironomidae sp. BIOUG20749-B02 / Chironomidae / Diptera	
111	(281/311)	90.4	MG144554 Chironomidae sp. BIOUG27558-D07 / Chironomidae / Diptera	
140	(280/310)	90.3	JN887059 Cricotopus bimaculatus / Chironomidae / Diptera	
149	(273/303)	90.1	KY833856 Megaselia sp. BIOUG02394-D11 / Phoridae / Diptera	
67	(282/313)	90.1	MN344567 Kamimuria tibialis / Perlidae / Plecoptera	
147	(281/312)	90.1	MW301815 Pilaria sp. ZS10 / Limoniidae / Diptera	
155	(275/306)	89.9	LC057190 Drosophila trilutea / Drosophilidae / Diptera	
343	(281/313)	89.8	EU678468 Leohumicola sp. DAOM 239516 / /	fungus
162	(280/312)	89.7	LC329265 Rheotanytarsus tamaquintus / Chironomidae / Diptera	
269	(280/312)	89.7	MN362995 Lebertia sp. BIOUG15123-B05 / Lebertiidae / Trombidiformes	Acariformes
273	(280/312)	89.7	MF744731 Hydryphantes sp. BIOUG18156-B10 / Hydryphantidae / Trombidiformes	Acariformes
138	(279/311)	89.7	MG301788 Orthocladiinae sp. BIOUG23941-E02 / Chironomidae / Diptera	
165	(279/311)	89.7	MF835529 Tachinidae sp. BIOUG21146-F12 / Tachinidae / Diptera	
350	(279/311)	89.7	MN593345 Arthrocladium fulminans / Trichomeriaceae / Chaetothyriales	fungus
158	(278/310)	89.7	KC263146 Antocha sp. SS-2012 / Limoniidae / Diptera	
220	(269/300)	89.7	MF881631 Drosophilinae sp. BIOUG24085-F09 / Drosophilidae / Diptera	
239	(277/309)	89.6	JQ907722 Goeracea genota / Goeridae / Trichoptera	
151	(274/306)	89.5	JN285990 Zavrelimyia sp. 1ES / Chironomidae / Diptera	
89	(280/313)	89.5	LC462308 Corynoneura lobata / Chironomidae / Diptera	
112	(280/313)	89.5	MG300230 Tanypodinae sp. BIOUG21865-B09 / Chironomidae / Diptera	
150	(280/313)	89.5	MF476250 Simulium atratum / Simuliidae / Diptera	
262	(278/311)	89.4	MG895845 Sperchon glandulosus / Sperchontidae / Trombidiformes	Acariformes
51	(261/292)	89.4	MG516460 Chromarcys magnifica / Oligoneuriidae / Ephemeroptera	
318	(269/301)	89.4	JN418688 Pinnularia sp. 9 CS-2011 / Pinnulariaceae / Naviculales	alga
108	(280/314)	89.2	KR457791 Orthocladius sp. BOLD-2016 / Chironomidae / Diptera	
153	(277/311)	89.1	KR438630 Nanocladius dichromus / Chironomidae / Diptera	
175	(267/300)	89.0	HM385606 Tanytarsus sp. TTDFW1048-09 / Chironomidae / Diptera	
300	(273/307)	88.9	FN601011 Ptochoecetis africana / Leptoceridae / Trichoptera	
172	(278/313)	88.8	LC495094 Benthalia sp. HM-2012 / Chironomidae / Diptera	
78	(277/312)	88.8	MK774407 Paragnetina sp. OPU_BS_2018-050-TM-PL / Perlidae / Plecoptera	
156	(277/312)	88.8	FM998448 Cheumatopsyche persica / Hydropsychidae / Trichoptera	
274	(277/312)	88.8	MW369218 Sperchonopsis aff. verrucosa VZ19076 / Sperchontidae / Trombidiformes	Acariformes
226	(276/311)	88.7	JX448423 Parascythopus exsulans / Curculionidae / Coleoptera	Parascythopus intrusus (Kôno, 1948)
247	(273/308)	88.6	JQ519820 Chaetogaster diastrophus / Naididae / Haplotaxida	Oligochaeta
270	(271/306)	88.6	MF458740 Lebertia porosa / Lebertiidae / Trombidiformes	Acariformes
249	(270/305)	88.5	GQ355367 Chaetogaster diastrophus / Naididae / Haplotaxida	Oligochaeta
83	(277/313)	88.5	LC462302 Corynoneura tenuistyla / Chironomidae / Diptera	
163	(277/313)	88.5	KT613493 Tanytarsus tamaduodecimus / Chironomidae / Diptera	
178	(277/313)	88.5	KC750520 Riethia stictoptera / Chironomidae / Diptera	
268	(276/312)	88.5	MW369264 Torrenticola aff. amplexa VZ19145 / Torrenticolidae / Trombidiformes	Acariformes
84	(268/303)	88.4	KF489833 Corynoneura mediaspicula / Chironomidae / Diptera	
193	(260/294)	88.4	MT262583 Simulium sp. BPD1 / Simuliidae / Diptera	
330	(274/310)	88.4	KC791166 Neoporphyra haitanensis / Bangiaceae / Bangiales	red alga
169	(278/315)	88.3	JF287884 Rheotanytarsus sp. BOLD:AAH3855 / Chironomidae / Diptera	
346	(270/306)	88.2	KY318514 Pseudogymnoascus destructans / Pseudeurotiaceae /	fungus
24	(262/297)	88.2	MH823334 Proepeorus nipponicus / Heptageniidae / Ephemeroptera	Epeorus nipponicus (Ueno,1931)
176	(276/313)	88.2	MN150323 Chironomidae sp. CH08 Zhangqiao Village 3 / Chironomidae / Diptera	
267	(276/313)	88.2	KJ709343 Arrenurus sp. BOLD:ACH9257 / Arrenuridae / Trombidiformes	Acariformes
348	(275/312)	88.1	KR704425 Verticillium nonalfalfae / Plectosphaerellaceae / Glomerellales	fungus
229	(267/303)	88.1	GU711742 Himalopsyche sp. XZ CN1 / Rhyacophilidae / Trichoptera	
49	(270/307)	87.9	MK403745 Ablabesmyia monilis / Chironomidae / Diptera	
63	(276/314)	87.9	AB770139 Oyamia lugubris / Perlidae / Plecoptera	
152	(268/305)	87.9	KY861853 Dictenidia leigongshanensis / Tipulidae / Diptera	
80	(275/313)	87.9	MH840672 Sweltsa borealis / Chloroperlidae / Plecoptera	
174	(266/303)	87.8	KP462171 Chironomidae sp. WHW-2015 / Chironomidae / Diptera	
349	(263/300)	87.7	MN593345 Arthrocladium fulminans / Trichomeriaceae / Chaetothyriales	fungus
282	(270/308)	87.7	HM377216 Hydryphantes sp. KOWMC070-09 / Hydryphantidae / Trombidiformes	Acariformes
52	(268/306)	87.6	LC329151 Orthocladius kanii / Chironomidae / Diptera	
272	(275/314)	87.6	MW369201 Protzia caucasica / Hydryphantidae / Trombidiformes	Acariformes
356	(274/313)	87.5	KU168424 Cairneyella variabilis / Helotiaceae / Helotiales	fungus
192	(273/312)	87.5	KU373672 Ceratopogon sp. BOLD:ACI9186 / Ceratopogonidae / Diptera	
221	(272/311)	87.5	HQ938470 Ordobrevia nubifera / Elmidae / Coleoptera	
265	(265/303)	87.5	KU243801 Testudacarus harrisi / Torrenticolidae / Trombidiformes	Acariformes
219	(270/309)	87.4	KT818884 Hydora sp. EJD-2015 / Elmidae / Coleoptera	
195	(256/293)	87.4	KP252653 Simulium travisi / Simuliidae / Diptera	
345	(268/307)	87.3	KX257489 Cladophialophora bantiana / Herpotrichiellaceae / Chaetothyriales	fungus
62	(274/314)	87.3	AB770139 Oyamia lugubris / Perlidae / Plecoptera	
191	(273/313)	87.2	JF872386 Ceratopogonidae sp. BOLD:AAG6465 / Ceratopogonidae / Diptera	
359	(266/305)	87.2	MK637641 Sydowia polyspora / Dothioraceae / Dothideales	fungus
157	(273/314)	86.9	EU493666 Drosophila nigella / Drosophilidae / Diptera	
263	(272/313)	86.9	HQ938536 Sperchon sp. BOLD:AAN9213 / Sperchontidae / Trombidiformes	Acariformes
333	(269/310)	86.8	GU070901 invertebrate environmental sample / /	unspecified
232	(268/309)	86.7	MG511784 Rhyacophilidae sp. 10BBEPT-0215 / Rhyacophilidae / Trichoptera	
13	(273/315)	86.7	MH844244 Anopheles braziliensis / Culicidae / Diptera	
303	(265/306)	86.6	JN304924 Chrysauginae sp. BOLD:AAM6842 / Pyralidae / Lepidoptera	moth
50	(258/298)	86.6	MF668536 Siphlaenigma janae / Siphlaenigmatidae / Ephemeroptera	
14	(270/312)	86.5	KP970706 Rhithrogena tetrapunctigera / Heptageniidae / Ephemeroptera	
293	(249/288)	86.5	MN674851 Hydryphantidae sp. BOLD:ADS0542 / Hydryphantidae / Trombidiformes	Acariformes
190	(270/313)	86.3	MF105765 Culicoides longipennis / Ceratopogonidae / Diptera	
68	(263/306)	85.9	KR665902 Drosophila paramelanica / Drosophilidae / Diptera	
285	(269/313)	85.9	MW369236 Parathyas dirempta / Hydryphantidae / Trombidiformes	Acariformes
347	(268/312)	85.9	MN657181 Cladosporium sphaerospermum / Cladosporiaceae / Cladosporiales	fungus
278	(263/307)	85.7	MG319031 Sperchontidae sp. BIOUG22338-D12 / Sperchontidae / Trombidiformes	Acariformes
332	(268/313)	85.6	MT228925 Vexillifera sp. AKu-2020a / Vexilliferidae / Dactylopodida	Amoebozoa
284	(265/310)	85.5	MN364233 Parathyas sp. BOLD:AAM7957 / Hydryphantidae / Trombidiformes	Acariformes
358	(265/310)	85.5	KX011536 Zasmidium cellare / Mycosphaerellaceae / Mycosphaerellales	fungus
5	(264/309)	85.4	KJ675257 Cinygmula sp. JMW3 / Heptageniidae / Ephemeroptera	
82	(266/312)	85.3	KX576477 Taenionema atlanticum / Taeniopterygidae / Plecoptera	
251	(258/303)	85.1	GQ355374 Pristina aequiseta / Naididae / Haplotaxida	Oligochaeta
283	(263/309)	85.1	KC263080 Sperchon sp. SS-2012 / Sperchontidae / Trombidiformes	Acariformes
4	(267/314)	85.0	MN961316 Heptageniidae sp. OPU BS 2019-017-DB-EP / Heptageniidae / Ephemeroptera	
208	(260/306)	85.0	KR489256 Melanophthalma helvola / Latridiidae / Coleoptera	
280	(262/309)	84.8	MG835761 Axonopsis sp. 1BHL041917av / Aturidae / Trombidiformes	Acariformes
254	(261/308)	84.7	LN999074 Enchytraeidae sp. 1 RV-2016 / Enchytraeidae / Enchytraeida	Oligochaeta
207	(266/314)	84.7	MN640425 Cloeon perkinsi / Baetidae / Ephemeroptera	
206	(265/313)	84.7	JQ662499 Baetodes sp. JMW1 / Baetidae / Ephemeroptera	
259	(265/313)	84.7	KP845499 Harpacticoida sp. BOLD:ACM2620 / / Harpacticoida	copepod
222	(257/304)	84.5	FJ819961 Anacaena sp. 5 MTM-2009 / Hydrophilidae / Coleoptera	
264	(267/316)	84.5	MF746983 Sperchontidae sp. BIOUG18716-A05 / Sperchontidae / Trombidiformes	Acariformes
201	(264/313)	84.3	MT830952 Labiobaetis sabordoi / Baetidae / Ephemeroptera	
253	(260/309)	84.1	GU014012 Megascolecidae sp. DPEW46886 / Megascolecidae / Crassiclitellata	Opisthopora
363	(233/277)	84.1	HQ923626 Phrixocomes sp. ANIC7 / Geometridae / Lepidoptera	moth
189	(254/302)	84.1	KX453756 Liponeura cordata / Blephariceridae / Diptera	
311	(259/308)	84.1	MN356524 Oribatella sp. BOLD:AAL8123 / Oribatellidae / Sarcoptiformes	Acariformes
6	(253/301)	84.1	HQ261141 Paraleptophlebia sp. AMI 1 / Leptophlebiidae / Ephemeroptera	
321	(252/300)	84.0	MT222521 Globisporangium spinosum / Pythiaceae / Pythiales	fungus
204	(262/312)	84.0	KF563030 Baetis thermicus / Baetidae / Ephemeroptera	
367	(262/312)	84.0	MW369294 Atractides tener / Hygrobatidae / Trombidiformes	Acariformes
73	(261/311)	83.9	MK132423 Nemoura sp. 3 MG-2018 / Nemouridae / Plecoptera	
203	(261/311)	83.9	MH841573 Baetis bicaudatus / Baetidae / Ephemeroptera	
317	(239/285)	83.9	EF164936 Sellaphora pupula / Sellaphoraceae / Naviculales	alga
319	(258/308)	83.8	MF997422 Nitzschia alba / Bacillariaceae / Bacillariales	alga
290	(262/313)	83.7	LR827697 Agraylea multipunctata / Hydroptilidae / Trichoptera	
289	(241/288)	83.7	MW369278 Lebertia porosa / Lebertiidae / Trombidiformes	Acariformes
69	(256/306)	83.7	KR665902 Drosophila paramelanica / Drosophilidae / Diptera	
304	(261/312)	83.7	KX296397 Hydroptilidae sp. TVTRI0057 / Hydroptilidae / Trichoptera	
275	(255/305)	83.6	MN359216 Mideopsis sp. BOLD:AAL8123 / Mideopsidae / Trombidiformes	Acariformes
357	(260/311)	83.6	EU678467 Myxotrichum deflexum / Myxotrichaceae /	fungus
324	(137/164)	83.5	KR879316 Pholetesor sp. BOLD-2016 / Braconidae / Hymenoptera	wasp
331	(246/295)	83.4	KR665197 Mycetophilidae sp. BOLD-2016 / Mycetophilidae / Diptera	
32	(256/307)	83.4	JQ661610 Ephemerella dorothea / Ephemerellidae / Ephemeroptera	
256	(264/317)	83.3	KY633394 Branchiodrilus hortensis / Naididae / Haplotaxida	Oligochaeta
217	(259/311)	83.3	KR561360 Saldidae sp. BOLD-2016 / Saldidae / Hemiptera	shore bug
281	(259/311)	83.3	JX838402 Hydryphantes sp. BOLD:AAF4149 / Hydryphantidae / Trombidiformes	Acariformes
320	(251/302)	83.1	KF768027 Dimeregramma acutum / Plagiogrammaceae / Triceratiales	alga
327	(187/225)	83.1	MT925539 Protancylus adhaerens / Protancylidae / Hygrophila	Gastrapoda
23	(260/313)	83.1	KM570892 Dolichopodidae sp. BOLD:AAG9626 / Dolichopodidae / Diptera	
198	(259/312)	83.0	HG935109 Cloeon cf. smaeleni GL30 / Baetidae / Ephemeroptera	
276	(259/312)	83.0	MG316728 Mideopsis sp. BIOUG23251-F06 / Mideopsidae / Trombidiformes	Acariformes
286	(259/312)	83.0	MG449836 Pionidae sp. BIOUG30216_F02 / Pionidae / Trombidiformes	Acariformes
279	(261/315)	82.9	MW369209 Lebertia aff. inaequalis VZ19067 / Lebertiidae / Trombidiformes	Acariformes
335	(251/303)	82.8	MH124198 Acanthamoeba sp. / Acanthamoebidae / Longamoebia	Amoebozoa
291	(260/314)	82.8	HQ563462 Micropterix schaefferi / Micropterigidae / Lepidoptera	moth
36	(259/313)	82.7	KM954873 Dolichopodidae sp. BOLD:ACB1149 / Dolichopodidae / Diptera	
202	(258/312)	82.7	KR134645 Baetodes sp. gmycM20 / Baetidae / Ephemeroptera	
292	(258/312)	82.7	MG311338 Lebertia sp. BIOUG25361-C07 / Lebertiidae / Trombidiformes	Acariformes
316	(243/294)	82.7	MH681084 Pinnularia macilenta / Pinnulariaceae / Naviculales	alga
277	(238/288)	82.6	MG315676 Hydrodroma sp. BIOUG23251-F10 / Hydrodromidae / Trombidiformes	Acariformes
266	(257/311)	82.6	JN018109 Torrenticola amplexa / Torrenticolidae / Trombidiformes	Acariformes
241	(244/296)	82.4	KX842664 Platysticta serendibica / Platystictidae / Odonata	
312	(244/296)	82.4	MN674518 Oppiidae sp. BOLD:AAL7979 / Oppiidae / Sarcoptiformes	Acariformes
288	(229/278)	82.4	KF966651 Penthaleidae sp. Q080 / Penthaleidae / Trombidiformes	Acariformes
44	(252/306)	82.4	HE651331 Compsoneuria sp. 6 LV-2012 / Heptageniidae / Ephemeroptera	
43	(261/317)	82.3	KY262520 Serratella ignita / Ephemerellidae / Ephemeroptera	
42	(247/300)	82.3	KJ964059 Melanophila acuminata / Buprestidae / Coleoptera	
37	(255/311)	82.0	KX038172 Zephlebia pirongia / Leptophlebiidae / Ephemeroptera	
366	(112/137)	81.8	KY698160 Cladius grandis / Tenthredinidae / Hymenoptera	wasp
260	(250/307)	81.4	MH976580 Eoschizopera sp. n. aff. syltensis SR-2019 / Miraciidae / Harpacticoida	copepod
338	(245/301)	81.4	EU681393 Bachelotia antillarum / /	alga
75	(244/300)	81.3	KY428239 Hexachaeta eximia / Tephritidae / Diptera	
328	(230/283)	81.3	MT380098 Megastigmus sp. 8 NHL-2020 / Megastigmidae / Hymenoptera	wasp
35	(245/302)	81.1	MN961290 Ephemera strigata / Ephemeridae / Ephemeroptera	
313	(247/306)	80.7	MN348626 Mucronothus sp. BOLD:AAL6084 / Trhypochthoniidae / Sarcoptiformes	Acariformes
258	(244/303)	80.5	MN745842 Ctenocalanus citer / Calanidae / Calanoida	copepod
325	(225/281)	80.1	KY492523 Stelligera rigida / Hemiasterellidae / Tethyida	sponge
314	(224/280)	80.0	HQ948517 Elachista discina / Elachistidae / Lepidoptera	moth
326	(238/299)	79.6	KR245768 Phronia sp. BOLD:ACL1048 / Mycetophilidae / Diptera	
257	(241/303)	79.5	MG936104 Harpacticoida sp. 11AlgonqNJ0018 / / Harpacticoida	copepod
261	(241/304)	79.3	KM611754 Cyclopoida sp. BOLD:AAG9785 / / Cyclopoida	copepod
287	(241/304)	79.3	MG320138 Tiphys sp. 09PROBE-02368 / Pionidae / Trombidiformes	Acariformes
309	(241/307)	78.5	MF917435 Suctobelbidae sp. BIOUG20423-H02 / Suctobelbidae / Sarcoptiformes	Acariformes
329	(243/310)	78.4	MK833920 Demospongiae sp. DGM-2019 / /	sponge
310	(246/314)	78.3	MN356725 Ceratoppia sp. BOLD:ADH9191 / Ceratoppiidae / Sarcoptiformes	Acariformes
315	(242/309)	78.3	KX651129 Dendrobaena octaedra / Lumbricidae / Crassiclitellata	Oligochaeta
368	(217/279)	77.8	KX281460 Stigmella sp. FicusauriculataVietnam / Nepticulidae / Lepidoptera	moth
364	(232/299)	77.6	HQ708994 Pythium vanterpoolii / Pythiaceae / Pythiales	fungus
323	(224/293)	76.5	HM033152 Rhodymenia stenoglossa / Rhodymeniaceae / Rhodymeniales	red alga
365	(221/295)	74.9	MN116495 Beybienkonus acuticercus / Corydiidae / Blattodea	cockroach
369	(217/302)	71.9	KY264161 Thaumamermis zealandica / Mermithidae / Mermithida	nematoda

# Species showing high similarity scores (>95%) but not recorded from Japan.

## 2 Experimental Design, Materials and Methods

### 2.1 Collection of specimens

Benthic organisms were collected using a hand net (mesh size: 200 per inch) in a perturbed riverbed by kicking at two sampling stations: ARD (on 17 March 2017) and SST (on 29 June 2017). Sampling was performed in the run environment (slow riffle) of both stations ARD and SST, and in rapid environment of SST (three community samples in total). The specimens were immersed in 70% ethanol until DNA was isolated. DNA of the three bulk community samples (one from ARD and two from SST) were separately extracted using conventional proteinase K treatment/phenol extraction/ethanol precipitation, as previously described [3].

### 2.2 DNA sequencing

The DNA libraries for sequencing were constructed by 2-step tailed PCR. The first PCR amplifications of the target gene (partial coding region of *cox1*) were performed using PrimeSTAR® HS DNA Polymerase (Takara Bio Co. Ltd., Kusatsu, Japan) for 35 cycles of 98°C-10 s / 40°C-5 s / 72°C-60 s with an initial incubation at 95°C for 1 min and a final elongation at 72°C for 7 min, using a set of primers IntF and HCOm [2]. The nucleotide sequences of the primers (containing the linker for the second PCR) were as follows:

IntF, ACACTCTTTCCCTACACGACGCTCTTCCGATCTGGWACWGGWTGAACWGTWTAYCCYCC;
HCOm, GTGACTGGAGTTCAGACGTGTGCTCTTCCGATCTTAHACTTCNGGGTGKCCRAARAATCA.

The amplified fragments were purified by AMPure XP (Beckman Coulter, Brea, CA, USA). The second PCR was performed using ExTaq® DNA Polymerase (Takara Bio Co. Ltd.) for 12 cycles of 94°C-30 s / 60°C-30 s / 72°C-30 s with an initial incubation at 94°C for 2 min and a final elongation at 72°C for 5 min, with a set of DNA primers containing octanucleotide tag for indexing. The nucleotide sequences of primers were as follows:

2^nd^-F, AATGATACGGCGACCACCGAGATCTACAC-[octanucleotide tag]-ACACTCTTTCCCTACACGACGC;
2^nd^-R, CAAGCAGAAGACGGCATACGAGATTAGCTGCAGTGACTGGAGTTCAGACGTGTG.

Two technical replicates for ARD and three technical replicates for each of two SST samples were prepared with different tags. The tag sequences for DRR311861, DRR311862, DRR311863, DRR311864, DRR311865, DRR311866, DRR311867, and DRR311868 were TCGACTAG, TTCTAGCT, CCTAGAGT, GCGTAAGA, CTATTAAG, AAGGCTAT, GAGCCTTA, and TTATGCGA, respectively. Quality of the DNA libraries was assessed by Fragment Analyzer with dsDNA 915 Reagent Kit (Agilent Technologies, Santa Clara, CA, USA). High-throughput parallel sequencing was performed by Miseq (Illumina, San Diego, CA, USA) capable of generating 2 × 300 base paired-end reads. The library construction (after the first PCR) and sequencing were outsourced to Seibutsu Giken Co. Ltd. (Sagamihara, Japan) (https://gikenbio.com/).

### 2.3 Operational Taxonomic Units

Among the raw sequence reads (472,697 reads in total of DRR311861-DRR311868), reads with quality scores less than 20 were discarded, and those of the 313 bp net length of paired sequences were sorted out. Valid sequences (227,467 reads) were collapsed into 41,010 groups with 100% identity, and the groups consisting of less than three reads were discarded. Neighbouring groups with a single nucleotide difference were combined each other to form an OTU, and a sequence with the largest number of reads within the OTU was defined as the OTU representative sequence. A total of 888 OTUs were obtained and subjected to GMYC analysis. The R script used to produce OTUs and OTU representatives in FASTA format are deposited in Mendeley Data (DOI:10.17632/pfhf4kt37s.1).

**Fig. 1:**
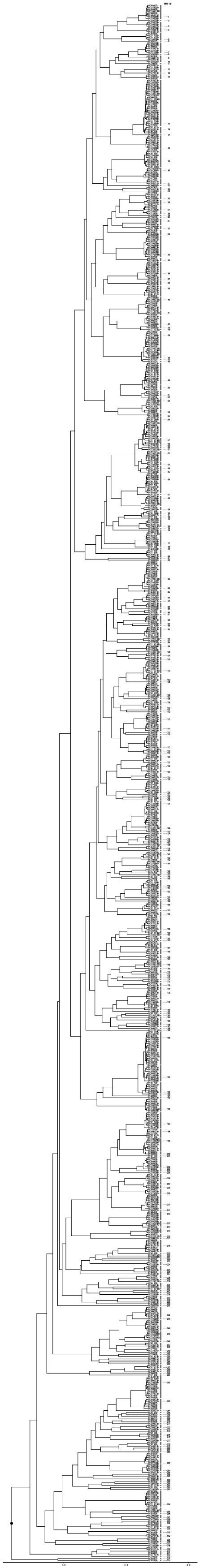
Species clusters estimated by GMYC. Each vertical bar indicates a cluster of OTUs or singleton assigned to single GMYC species. OTUs are numbered arbitrarily, and the numbers in parentheses after OTU names indicate the number of reads of OTUs. Numbers with dotted lines refer to GYMC IDs. Owing to the size of the phylogenetic tree, Fig. 1 is divided into 1a and 1b, both of which are linked at the node indicated by the closed circle (●).

**Fig. 1:**
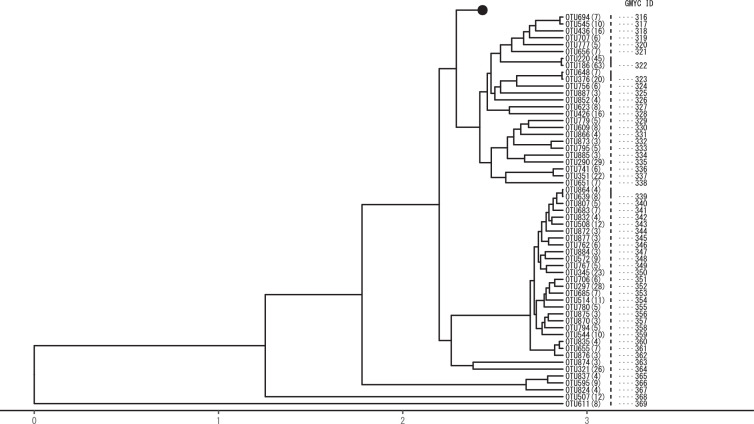
Continued

### 2.4 Estimation of GMYC species

A phylogenetic tree of 888 OTUs was reconstructed using Markov chain Monte Carlo (MCMC) methods by MrBayes 3.2.7a with the GTR+I+G molecular clock model. GMYC species boundaries were estimated using SPLITS package in R.

## CRediT authorship contribution statement

**Kei Wakimura:** Investigation, Writing – original draft. **Koji Inai:** Software, Investigation. **Kazumi Tanida:** Resources, Writing – review & editing. **Kozo Watanabe:** Conceptualization, Writing – review & editing, Funding acquisition. **Mikio Kato:** Project administration, Conceptualization, Resources, Writing – review & editing, Funding acquisition.

## Declaration of Competing Interest

The authors declare that they have no known competing financial interests or personal relationships that could have appeared to influence the work reported in this paper.

## Data Availability

Operational taxonomic units of cox1 amplified from bulk community DNA samples in Takamigawa River, Nara, Japan (Original data) (Mendeley Data). cox1 amplicon sequences of riverine metagenomes (Original data) (DDBJ (DNA Data Bank of Japan)).
